# p53 Isoforms and Their Implications in Cancer

**DOI:** 10.3390/cancers10090288

**Published:** 2018-08-25

**Authors:** Maximilian Vieler, Suparna Sanyal

**Affiliations:** Department of Cell and Molecular Biology, Uppsala University, Box-596, BMC, Uppsala SE-75124, Sweden; max.vieler@gmail.com

**Keywords:** p53, cancer, p53 isoform, ∆133p53, ∆160p53, ∆40p53, aggregation, prion

## Abstract

In this review we focus on the major isoforms of the tumor-suppressor protein p53, dysfunction of which often leads to cancer. Mutations of the *TP53* gene, particularly in the DNA binding domain, have been regarded as the main cause for p53 inactivation. However, recent reports demonstrating abundance of p53 isoforms, especially the N-terminally truncated ones, in the cancerous tissues suggest their involvement in carcinogenesis. These isoforms are ∆40p53, ∆133p53, and ∆160p53 (the names indicate their respective N-terminal truncation). Due to the lack of structural and functional characterizations the modes of action of the p53 isoforms are still unclear. Owing to the deletions in the functional domains, these isoforms can either be defective in DNA binding or more susceptive to altered ‘responsive elements’ than p53. Furthermore, they may exert a ‘dominant negative effect’ or induce more aggressive cancer by the ‘gain of function’. One possible mechanism of p53 inactivation can be through tetramerization with the ∆133p53 and ∆160p53 isoforms—both lacking part of the DNA binding domain. A recent report and unpublished data from our laboratory also suggest that these isoforms may inactivate p53 by fast aggregation—possibly due to ectopic overexpression. We further discuss the evolutionary significance of the p53 isoforms.

## 1. p53—The Gene, the Protein, and the Isoforms

The tumor-suppressor protein p53 has been named the ‘guardian of the genome’ [1], as well as the ‘coordinator of the underlying processes of the hallmarks of cancer’ [2]. This is because its inactivation paves the way for cancers. p53 is a DNA binding protein which halts the cell cycle upon genomic stress [3] and essentially hinders proliferation of the cells with damaged DNA [4]. Upon DNA damage, p53-dimers bind to the p53-responsive elements (referred hereafter as RE), forming a dimer of dimers [5], initiating transcription of p53 responsive genes, and ultimately triggering the p53 pathway. The p53 pathway is involved in cell apoptosis, cell cycle arrest or DNA repair [3]. The main players of the p53 pathway are the mouse double minute protein 2 (MDM2) and the protein p14ARF. MDM2 is encoded by the *MDM2* gene; it targets p53 for proteasomal degradation [6]. p14ARF is encoded by the *ARF* gene, which inhibits MDM2 and raises p53 levels [7]. Additionally, expression of p53 isoforms can alter the transcriptional targets of p53 [8,9].

p53 research is closely connected to clinical applications as an indicator for cancer types. The knowledge acquired from p53 research allows prediction of the possible outcomes of various cancers, and also aids in directing treatments by the use of specific compounds [10]. Since the early 1990s, the field of study of p53 has generated an ocean of information which is instrumental in understanding cancer. The next step is to discover the treatment of dysfunctional p53 itself. In order to do so, we need to understand the principles and the underlying mechanisms for formation of the p53 isoforms and their molecular interactions. 

Because it controls many cell-fate-deciding genes, p53 has a prominent role in cancer—both for the diagnosis and the treatment. Inactivation of p53 leads to malicious apoptosis. In simple words, the cells with DNA damage, whose normal fate is to die, can survive, divide irregularly, and cause cancer. There are many ways how p53 can be inactivated. Mutations in p53’s DNA binding domain (DBD) are the major cause of its inactivation. This makes the protein impaired to bind to the target DNA [11]. These mutations are widely studied and can be directly correlated with diseases. Other than the point mutations, various truncated forms of p53, commonly called as p53 isoforms, can also be responsible for its inactivation. The p53 isoforms are the main focus of this review and we discuss in detail their constitution, potential mechanisms of action, and the evolutionary significance for their creation.

### 1.1. The TP53 Gene and the Synthesis of the p53 Isoforms

**Figure 1:**
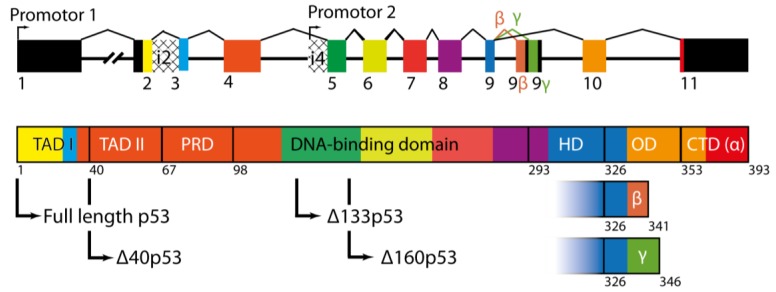
(**Top**) Canonical exons (boxes) and alternative 5′- untranslated regions (UTRs) (checked boxes) of the *TP53* gene. The colored exons code for different domains of the p53 protein. Promoter 1 produces a transcript which translates to the full-length p53 (FLp53) and the ∆40p53 isoform; the latter is translated only if intron-2 is retained in the transcript. Promoter P2 produces a transcript coding for ∆133 and ∆160p53 isoforms starting from the 133th and 160th codons. The C-terminal isoforms of p53 (α, β, and γ) are controlled by alternative splicing of the exon 9. (**Bottom**) Different domains of the FLp53 and their correspondence with the exons (shown with the same color code) of the *TP53* gene: transactivation domain I (TAD I); transactivation domain II (TAD II); proline rich domain (PRD); DNA-binding domain; hinge domain (HD) oligomerization domain (OD) C-terminal domain (CTD). The arrows indicate the start point (N-terminus) of the particular isoform and determine the domains included in the respective isoform. Bottom right: The open boxes represent the two other C-terminal isoform-variants β and γ.

The *TP53* gene is spread over 13 exons (Figure 1) located on the human chromosome 17p13.1 [12]. Using multiple promotors, alternative splicing, and the internal ribosome entry site (IRES), this gene can create 12 different isoforms of the p53 protein [13]. The isoform expression is regulated in the transcriptional level by alternative promoter usage and by alternative splicing of intron-2 and intron-9.

Due to the presence of two promotors, P1 and P2, *TP53* is often referred to as a dual gene [13]. The duality originates from the two promotors P1 and P2, leading to different isoforms of p53. The promotor P1 transcribes an mRNA piece which can translate either the full-length p53 (FLp53) or the ∆40p53 isoform starting with codon 40. In the latter case, the FLp53 transcript retains intron-2 and the ∆40p53 isoform synthesizes from an internal ribosome entry site (IRES) [14,15]. The ∆40p53 isoform can also be formed from the ∆40p53 transcript, which has the intron-2 spliced out from the p53 transcript. The ∆133 and ∆160 isoforms are transcribed from the promotor P2, starting with the methionine at codon 133 or 160, respectively.

Besides these, three other isoforms of p53 show variations in the C-terminus. These isoforms, named α, β, and γ, are the results of alternative splicing of the exon 9 (Figure 1). The α isoform retains all the exons including exons 10 and 11, which translate the hinge and the oligomerization domain of p53. Alternative splicing of the exon 9 results in the exon 9β or the exon 9γ; these transcripts after translation lead to the β and γ isoforms of p53. In both cases, the exons have stop codons, which will lead to the termination of translation, causing C-terminal truncation of varying lengths (Figure 1).

### 1.2. p53 Protein and Its Different Domains

p53 is a tetrameric protein which commonly tetramerizes with four identical subunits and acts as a transcription factor. The monomers combine into two dimers, which bind to a consensus DNA sequence, referred as RE [16,17]. However, the p53 structure on DNA can be much more complex than mere tetramers [18,19]. Using electron microscopy, it has been demonstrated that the p53 tetramers, by virtue of interaction between two distant p53 REs, can stack on each other and cause DNA looping. 

**Figure 2:**
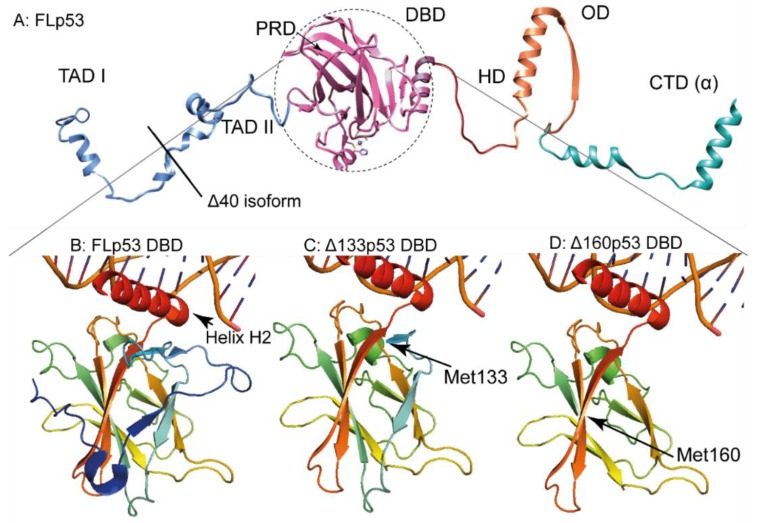
FLp53 and its isoforms ∆40, ∆133, and, ∆160. (**A**) A schematic representation of FLp53α with its five domains clearly designated: the N-terminal domain with transactivation domains I and II (TADI and TADII; blue), the DNA binding domain (DBD; pink), the hinge domain (HD; red), the oligomerization domain (OD; orange) and the carboxy-terminal regulatory domain (CTD; green). The NTD structure is assembled by superimposition of pdb ID 2K8F (Chain B, residues 1–35) and 2B3G (chain B, residues 35–56). The DBD and OD were derived from pdb ID 3TS8. The model is created in Chimera. The start of the ∆40p53 isoform is indicated by a black line. (**B**–**D**): Close-up on the DBD in FLp53 and its two isoforms modeled in Pymol based on pdb ID 3TS8. Figures demonstrate how the shorter isoforms ∆133p53 and ∆160p53 lack β-sheet stabilizing strands. B: DBD of the FLp53. C: DBD of the ∆133p53 isoform starting with Met^133^. D: DBD of the ∆160p53 isoform starting with Met^160^.

p53 itself has a modular structure comprising of six domains (Figure 1): the two transactivation domains I and II (TAD I, and TAD II), spanning residues 1–67; the proline-rich region (PRD) with residues 68–98; the DNA-binding domain (DBD, also called the core domain) with residues 94–292; the hinge domain (HD), with residues 293–325; the oligomerization domain (OD) with residues 326–353; and the carboxy-terminal regulatory domain (CTD) spanning residues 353–393 [20]. Figure 2A represents a realistic model of FLp53 with all domains.

The N-terminus of FLp53 is largely disordered (Figure 2A). The two N-terminal transactivation domains TAD I (residues 1–40) and TAD II (residues 41–67) can independently activate transcription of the target genes [21]. These TADs are not equivalent and are required to induce different promotors [22]. The natively unfolded TADs [23] interact with a variety of proteins, transmitting the binding event. These proteins include transcription factor II D and II A (TFIID, TFIIA), the TATA box binding protein (TBP), mouse double minute 2 homolog (MDM2), and the histone acetyltransferase p300-CREB-binding protein coactivator family (CBP/p300) [24,25,26]. The N-terminus also helps to recruit the chromatin remodeling factors, which facilitate the access of p53 and the basal transcription machinery to DNA [4].

The proline-rich domain or PRD (residues 68–98) links TAD to DBD with 12 proline residues (Figure 1 and Figure 2A); this domain includes four copies of the PXXP motif [27]. The PXXP motif constitutes a binding site for Src-Homology-3 domains, which mediates protein–protein interaction in signal transduction [28]. The PRD is required for apoptosis and growth suppression triggered by p53 [29].

The DNA-binding domain (DBD) (residues 94–292) (Figure 1 and Figure 2A) recognizes and binds specifically to a double-stranded DNA consensus site called ‘response element’ (referred hereafter as RE), which contains the decameric motif Pu-Pu-Pu-C-(A/T)|(T/A)-G-Py-Py-Py (Pu: purine, Py: pyrimidine) [30]. The DBD adopts an immunoglobulin-like β-sandwich fold including four cysteine boxes, which builds the crucial scaffold for DNA binding. The DBD binds to the major groove of the DNA with the highly-conserved long helix H2 (residues 270–286) [31] by an induced fit mechanism [32]. It has been hypothesized that the DBD can hop-on and hop-off the DNA in the search for REs, which has been further corroborated by molecular dynamics simulation [33]. DNA binding is facilitated by interactions between the DBD and the oligomerization domain (OD) [33].

Similar to the N-terminus, the whole C-terminal region of p53 (residue 347–393) is also predominantly unstructured with only few α-helices separated by random coil regions [11] (Figure 2A). The OD (residues 326–353) forms dimers through their helices, which in turn form the tightly packed tetramers [17]. The OD also contains the nuclear export signal (NES), which is masked by tetramerization of p53. Masking the NES prevents p53 from exporting to the cytoplasm, where p53 cannot regulate gene expression [34]. The OD helps to deform the bound DNA and thereby facilitates DBD for stable binding of DNA [33]. It is interesting to note that all N-terminal variants or isoforms of p53 contain the OD; which can be crucial for their functionality.

The C-terminal domain (CTD) (residues 353–393) controls the structure and function of the entire protein [35]. Similar to the N-terminal domain (NTD), it is too intrinsically disordered [36], and contains the site of post-translational modifications [35]. The CTD recognizes and binds to damaged DNA by a nonspecific DNA-binding site [37]. The DNA-CTD binding is based on low-affinity electrostatic interactions between the DNA and the lysine residues of CTD [35].

In the absence of DNA, p53 arranges to an open cross-shaped structure, with its loosely-coupled dimers interacting through the core domain. Upon DNA binding, the structure rigidifies and becomes more compact [35].

### 1.3. p53 Isoforms Are Variants of p53 with N- and C-Terminal Truncations

To date, 12 isoforms of p53 have been reported [13]; all are truncated forms of the FLp53. Based on whether the truncations are in the N- or the C-terminus of the protein, the isoforms have different nomenclature. The isoforms with N-terminal deletion are named according to the extent of deletion. These are mainly the ∆40p53, ∆133p53, and ∆160p53 isoforms, which lack various lengths from the N-terminus of FLp53, starting with methionine 40, 133, and 160, respectively (Figure 1). These N-terminal isoforms are the main focus of this review. The other isoforms show variation in the C-terminus and are named as α (full-length), β (52 amino acids shorter), and γ (47 amino acids shorter) (Figure 1). In fact, the FLp53 and all the three N-terminal isoforms can exist in α, β, and γ form.

The p53 isoforms bear definitive clinical relevance [12]. It has been observed that the p53 isoforms are differentially expressed in normal and tumorigenic tissue in different types of cancer [13]. Furthermore, several studies have reported abnormal expression of a particular p53 isoform in relation to a specific cancer (see Table 1). Traditionally, frequent mutations of the *TP53* gene have been regarded as mainly responsible for cancer [38]. However, different reports have shown that cancer can occur despite a low mutation rate of *TP53*; in many such cases a significant change in the expression level of the p53 isoforms has been observed. For instance, in breast cancer, with a mere 25% mutation rate, the expression of the p53 isoforms is largely altered. There are reports of decrease in the expression of the p53β- and p53γ isoforms by 60% and an increase in the expression of ∆133p53 by 40% [39]. The same pattern is observable in acute myeloid leukemia, where only 10% of the cases bear a mutation of *TP53*, while a large shift in the expression level of the p53β and p53γ isoforms can be observed [40]. These observations have opened up new perspectives for understanding disease manifestation in cancer and importantly, the role of the p53 isoforms in it. Understanding the mechanisms of action of the p53 isoforms can be instrumental for further improvement of cancer treatment. As of now, most of the correlations of abnormal expression of p53 isoforms and cancer development are case-dependent. More data is needed for generalization, but moreover, the underlying mechanism of action of the p53 isoforms in the normal cell needs be understood with molecular detail.

## 2. Major N-Terminal Isoforms of p53

The p53 isoforms were first identified in humans and mice by Matlashewski et al. (1984) [41] and Wolf et al. (1985) [42]. The expression of the isoforms is governed by alternative promoter usage and by alternative splicing (Figure 1) [13]. The isoforms mediate different cellular responses towards various stimuli and stresses [4]. The p53 isoforms, depending on the cellular environment, can cooperate with FLp53 to fine-tune the p53 pathway. Alternatively, the N-terminal isoforms can also inhibit the functions of FLp53 [43,44]. Thus, detailed understanding of the mode of action of the p53 isoforms is timely and indeed crucial in the field of cancer biology. If the correlation between the expression of the p53 isoforms and the onset of cancer can be understood, this knowledge can be used for prognosis of cancer combined with the knowledge of p53 mutations [12].

### 2.1. The ∆40p53 Isoform

The ∆40p53 isoform starts from the 40th codon of the *TP53* gene. Compared to all other N-terminal p53 variants, the ∆40p53 isoform bears the shortest truncation. Hence, it maintains most of the functions of the FLp53 (Figure 2A). As mentioned before, ∆40p53 can be translated by alternative splicing of the p53 transcript, when intron-2 is retained (Figure 1). This intron carries an in-frame stop codon, which causes termination of translation forming a very short peptide. An IRES exists following the stop codon, which allows re-initiation of translation at codon 40, which, being ATG, codes for methionine. Compared to the FLp53, this isoform lacks TAD I, but retains TAD II (Figure 2A). ∆40p53 can oligomerize with FLp53 in vivo and thereby regulate the function of FLp53 [45]. Moreover, because it retains only TAD II, ∆40p53 is capable of inducing a different set of p53-responsive genes than FLp53 [46]. It can induce many apoptosis-associated genes which are inaccessible by FLp53 [47]. In addition, ∆40p53α forms oligomers with a higher propensity than FLp53 [4] and small amounts of ∆40p53 can increase the transactivation effect of FLp53 [48]. Conversely, high ∆40p53 levels induce a different set of genes than the FLp53 [4]. The strong tendency of ∆40p53 to form oligomers demonstrates that small amounts of ∆40p53 can have a substantial effect on the p53 pathway [49]. In essence, ∆40p53 balances tissue regeneration and tumor suppression [50] while fine-tuning the p53 activity [4].

### 2.2. The ∆133p53 Isoform

The isoform ∆133p53 lacks 132 amino acids from the beginning of FLp53. The truncation includes both the TADs and part of DBD, and thus ∆133p53 imposes a more drastic effect on the p53 pathway. ∆133p53 can be produced in various ways. It can either be translated from the full p53 transcript or from a transcript starting from intron-2 or from intron-4 [12]. ∆133p53α lacks TAD I, TAD II, the PRD, loop L1 and the strands S2 and S2’ of the DBD [13].

Any change in the DBD must result into altered affinity and specificity to DNA, which is reflected by this isoform. Due to the absence of the amino acid K120, which is known to be crucial for DNA binding in FLp53 [5] ∆133p53α could be somewhat impaired in DNA-binding. However, other amino acids of DBD that are involved in DNA binding are still retained in this isoform. Moreover, ∆133p53 is capable of forming dimer or tetramer with FLp53 or other p53 isoforms carrying OD. Thus, most likely ∆133p53 binds to a different set of genes and acts in a context-dependent manner [51]. It has been demonstrated that ∆133p53 changes the promoter selectivity and transcriptional activity of the p53 transcription factor family (including p53/p63/p73 isoforms) to promote cell survival by inducing DNA repair [9] angiogenesis [52,53], and regulating immune response to tumor proliferation [54]. The ∆133p53 isoform also inhibits apoptosis by inducing expression of special sets of genes [55,56].

The DBD structure in ∆133p53 can be somewhat altered due to the loss of a stabilizing β-strand (Figure 2C). Furthermore, the loss of a part of the DBD may also affect the stability of the ∆133p53 isoform. Unpublished data from our laboratory demonstrated that ∆133p53 is highly prone to aggregate. In fact, ∆133p53 aggregates much faster than the FLp53 or p53C, an artificial p53 construct which comprises of only the DBD [57]. Hence, ∆133 could also exert its loss/alteration of function through aggregating with FLp53 and hindering FLp53 from binding the designated REs on the target DNA.

The exact mechanism of action of ∆133p53 is still unknown. It has been shown that ∆133p53α can rescue the cells from FLp53-induced apoptosis [56]. Alike ∆40p53α, this isoform can fine-tune the p53-dependent pathways [58], and regulate the cellular response to DNA damage [56]. ∆133p53α has been also connected to pro-angiogenesis in cancer [52]. Additionally, it can induce DNA double-strand break (DSB) repair [59]. ∆133p53α may delay the onset of replicative senescence and prolong the replicative lifespan, but it does immortalize or malignantly transform the cell [60]. In this way, ∆133p53α can rescue cells which have not been irreversibly damaged from apoptosis. Studies in zebrafish with the ∆133p53α-homolog showed that ∆113p53α-depleted embryos were susceptible to ionizing radiation. Restoring ∆113p53α rescued the development of the embryos by inducing p53-targeted gene expression [55].

### 2.3. The ∆160p53 Isoform

Initiating p53 translation from Met160 results in the smallest isoform—∆160p53α, which is also the most recently discovered isoform of p53 [61]. This isoform lacks both the TADs, the PRD, and the first conserved cysteine box of the DBD. However, it still retains the three other conserved cysteine boxes [12]. It has been shown that cells, which were previously thought to be p53-null, still express this isoform [62]. Since ∆160p53 lacks a major part of the DBD, it may have a molecular effect similar to mutant p53s [63]. Interestingly, frequent p53 mutations, such as R175H or R248Q, show elevated levels of expression of ∆160p53, suggesting that ∆160p53α isoform is widely involved in mutant p53’s ‘gain of function’ (GOF) feature [63]. Knocking-out of endogenous ∆160p53α inhibits R273H GOF and restores apoptosis. This suggests that the apoptosis inhibiting GOF of endogenous mutant p53 is dependent on ∆160p53α. Furthermore, ∆160p53α may induce mutant-like phenotypes, possibly to a greater extent than ∆133p53α [63]. It is still unclear whether ∆160p53α has specific genomic targets and can induce transcription, or if it simply acts by manipulating the efficacy of FLp53. Thus, this newly discovered p53 isoform introduces questions that requires dedicated investigation, especially on how it induces cancer.

## 3. Disease Perspective of the p53 Isoforms

A mutation in the *TP53* gene has been reported only in 25% of cancerous tumors [13], leaving many options for other factors that can influence p53 activity. Overexpression or dysregulation of p53 isoforms can account for the development of cancers, as they can inhibit the canonical p53 functions [39]. If p53 in inactivated, the DNA-damaged cells, which normally die, can survive and cause cancer. Clinical studies have found unusual expression of p53 isoforms in different cancers (see Table 1). The p53 isoforms can be seen as the intracellular microprocessors [58], as they fine-tune the p53 pathway in germ and somatic cells of vertebrates, enabling the vertebrate organism to maintain genomic stability [1] until maturity and even beyond [50]. All p53 isoforms have been detected in both cancer and normal tissues, except the ∆133p53β isoform, which had not been found in normal tissue as of 2010 [13]. Mutations and isoforms interact with each other and change the cancer outcome. The adverse effect of mutant p53 in ovarian cancer or in breast cancer is overcome by ∆133p53 or by p53γ, respectively [64,65]. The detailed disease perspective of the p53 isoforms is summarized in Table 1.

## 4. Ways to Inactivate or Modulate the p53 Pathway

Malfunction or inactivation of p53 leads to cancer [66]. The p53 pathway can be inactivated through different mechanisms. The most prominent and well-known mechanism is through missense mutation of p53, especially in its DBD, which alters p53’s DNA binding ability [48]. About 1200 individual mutations have been reported in p53 DBD in various cancers [67]. Other than mutations, p53 may also be inactivated by deletion of one or both p53 alleles, nonsense or splice site mutations in the OD, amplification of the *MDM2* gene, deletion of the ARF gene, or mislocalization of p53 to the cytoplasm [68]. Other mechanisms include the inactivation by viral proteins [69,70], and by bacterial infection [71]. Recent reports suggest that the N-terminally truncated isoforms of p53 also play important role in p53 inactivation. Some p53 mutations and isoforms change specificity to other genes, which modulate p53 function and may lead to ‘dominant negative effect’ or ‘gain of function’. Other than these mechanisms operating in the gene or the transcription level, aggregation of p53 and its isoforms can also be a way for p53 inactivation. The fact that p53 aggregates in a prion like fashion is a rather new discovery [57,72,73] and requires more investigation. These points are discussed below.

### 4.1. Mutations

Mutations result in a loss or modulation of transcriptional activity of the FLp53 and the obvious outcome is cancer [38,74,75]. Although cancer causing mutations can be found on almost all exons (2–11) encoding different domains of p53, the DBD or the core domain bears more than 80% of all missense mutations that are found in human cancers [11,76]. These mutations mostly affect/impair the DNA binding ability of p53. Some of these mutations act by affecting the structure, folding, or stability of the DBD, all of which result in altered sensitivity for DNA binding by p53 [11]. These are called ‘conformational p53 mutants’. Alternatively, some p53 mutants do not alter the structure of the DBD, yet hamper DNA binding partially or fully. These mutants are called ‘contact site mutants’ as the amino acids at the mutation sites interact directly with DNA in wild-type p53. In the DBD, six mutational hot-spots were identified. These are present at codons 175, 245, 248, 249, 273, and 282 [76]. Among these mutations, 175, 248, and 273 account for 20% of all somatic mutations [77,78]. In total, 25% of all p53 mutations lead to a translational frameshift that prematurely terminate the protein causing variations at the C-terminus. These are usually located outside of the exons 5–8, which code for the DBD [10]. In total, 4% of all mutations occur at the splice sites. These mutations affect regular intron splicing from the p53 transcript and may lead to various truncated or extended isoforms. The deletion mutations are more frequent in the N-terminus, which leads to ∆40p53, ∆133p53, and ∆160p53 isoforms [67].

p53 mutations are multifarious and hard to detect. Many missense mutations lead to a high level of heterogeneity in the protein functions [10]. Hence it is difficult to parallelize the relationship between mutations in p53 and the outbreak of cancer. Even less frequent mutations may occasionally result in severe effects on p53 function and eventually on the patient’s fate [79]. Hence, it is advisable to analyze all exons, from 2 to 11, for mutations. Cancers with high-frequency mutations (35–50%) are ovarian, esophageal, colorectal, head and neck, larynx, and lung cancers. Conversely, low-frequency mutation (20–35%) cancers are leukemia, sarcoma, testicular, and cervical cancer, and malignant melanoma [10]. These altogether make *TP53* the most frequently mutated gene in human cancers [79]. In fact, missense-mutated p53 leads to cancer with a much worse prognosis than with *TP53* deletions [74].

Many mutations may exert a ‘dominant negative effect’, inactivating wild-type p53’s DNA-binding ability by oligomerization [66,80]. Overall, the effect of mutant p53 is very case-dependent and unpredictable [38]. The activity of mutant p53 is heavily dependent on the type of tissue, pH-value, inflammation, active signaling pathways, and the interactions with stroma cell [81], and it is not always stable in all tumor cells [82]. Mutations can also lead to complications in cancer therapy; they can produce multidrug resistance [83]. Hence p53 mutations are considered to be even worse than a loss of function allele [74]. Moreover, mutations can result in a gain of function as discussed later [84].

### 4.2. Dominant Negative Effect

The dominant negative effect or DNE is an inhibitory effect exerted by the p53 mutants on the wild-type p53 proteins leading to cancer [85]. In simple words, the DNE prevents wild type p53 from exerting its canonical functions [86]. Indeed, data from 200 mutations show that DNE correlates with cancer frequency [66], and 160 out of 235 p53 mutations have been shown to exert a strong DNE in breast cancer. While carcinogenesis requires the loss of both alleles of the *TP53* gene, mutation of one allele of p53 can also result into loss of its function. One way by which the p53 mutants exert DNE on wild-type p53 is hetero-oligomerization [87,88]. This is not unexpected because most of the DBD missense mutants have no alteration in their OD [66]. The hetero-dimers/tetramers of mutant p53 with wild-type p53 probably target DNA sequences different from canonical ones.

While the hetero-oligomerization-based explanation may be relevant for DNE of the p53 conformational mutants, it cannot possibly explain the DNE exhibited by contact site mutants [87]. It has been proposed that these p53 mutants probably fail to transactivate certain p53 targets. Potentially, the p53 mutant/wild-type hetero-tetramers exhibit weaker affinity for DNA binding than the p53 homo-tetramers. This will have implications in expression levels of p53, regulated genes, and in turn, may cause DNE. Alternative theories suggest that the p53 mutants could be defective in binding to the transcriptional cofactors, which are essential for proper p53 activity.

### 4.3. Gain of Function

Missense-mutated p53 often exhibits a ‘gain of function’ (GOF) [81], which introduce neomorphic functions which are independent from the wild type p53 [86], and which lead to p53 phenotypes that are distinctly different from the p53 null cells [38]. The GOF is just not loss of function of wild-type p53. It is exerted by some p53 mutants, which exhibit a selective advantage to carcinogenesis by altering the patterns of gene expression [89].

Intriguingly, some mutant p53 are able to bind specific non-B-DNA structures, with a defect sequence-specific DNA binding ability [90]. These new and acquired functions [91] may include: (1) an earlier onset of more aggressive and metastatic tumors [92]; (2) cancer genomic instability i.e., an increased tendency of genome alteration during cell division [93,94]; (3) a replicative immortality with unlimited potential for cellular proliferation [95]; (4) sustained cell proliferation signaling that helps the cancers cells to evade the immune system and growth suppressors; (5) angiogenesis and lymphangiogenesis that leads to growth of new blood and lymphatic vessels [96]; and (6) reprogramming of energy metabolism, which enables the tumor draw more energy by facilitating blood vessel generation and increase in their glycolysis rate [97]. All these features are considered to be the emerging hallmarks of cancer. Missense mutations result in altered structures; these are known as conformational mutants. Alternatively, some mutations hamper DNA binding, which are known as contact mutants. The inability of the contact mutant p53s to bind directly to DNA hints that GOF must be mediated by other p53-binding proteins such as transcription factors or chromatin remodeling factors [81]. Furthermore, mutant p53 GOF may also be responsible for the expression of multi-drug resistance genes [98].

The GOF is not simply due to loss of function of wild-type p53. The p53 mutants actually alter the patterns of gene expression by hetero-oligomerization or by interacting with a different set of transcription factors. While the exact molecular basis for mutant p53 gain of function is still not clear, the ability of mutant p53 to exert oncogenic effects independently of wild-type p53 provides a selective advantage for p53 missense mutation in cancer. In fact, GOF can be a consequence of the previously discussed DNE exerted by the p53 mutants.

Similar to DNE, the role of the p53 isoforms in GOF is unclear. Since the contact mutants of p53 are defective in DNA binding and they are often associated with elevated expression of the p53 isoforms (e.g., Δ160p53 [63]), it is likely that the isoforms play a role in the process.

### 4.4. Aggregation

Aggregation of p53 might be a possible way to inactivate the p53 pathway, and it is even being proposed that, because of the formation of prion-like p53 amyloid aggregates, cancer could be regarded as prion disease [99,100]. p53 aggregates have been known for long [101]. Many in vivo and in vitro studies have demonstrated high potential of p53 to aggregate in both the amyloid and amorphous forms [57,102,103]. Upon misfolding, p53 oligomers were identified close to the nucleus, and p53 fibrils were located in the cytoplasm [104]. In vivo, p53 forms predominantly amorphous aggregates [72,73], but under certain conditions, p53 can also form amyloid fibrils [99]. Moreover, the p53-aggregation mechanism is different from the classical nucleation growth model [105]. A p53 amyloid can exhibit pathogenicity like any other amyloid disease [104]. In fact, prion-like aggregation of mutant p53 is correlated to the malfunction of p53 in cancer [103]. There are growing numbers of examples where p53 aggregates have been discovered in the cancerous tissues.

All three p53-domains can aggregate [72,106,107]. Despite this, the DBD has been the main subject for p53 aggregation studies [57,105]. Aggregation of p53 may be triggered by mutations and unfolding perturbations, which expose its core fragment, 251ILTIITL257 [108,109]. Mutant p53, has higher potential for aggregation [110] and can trigger the aggregation of wild type p53 [73], and even the aggregation of its paralogs p63 and p73 [111,112]. This co-aggregation of p53 and its paralogs may initiate interaction with new DNA-binding sites and thereby result into the p53 gain of function in cancer [84]. Alternatively, the dominant negative effect of the p53 mutants can be arising from their prion like behavior [110]. p53 aggregation might also raise cancer aggressiveness and progression [103].

Recently, we have demonstrated with fast kinetics that RNA from various source and sizes can modulate aggregation of p53c (the core domain or DBD) [57]. At low RNA to protein ratio, amorphous aggregation of p53 is facilitated. Conversely, at higher RNA to protein ratio, p53c aggregation is inhibited. In such conditions, amyloid oligomers of p53c are formed, which are capable of seeding fresh p53 solutions [57]. This finding, on one hand, points to more research in the internal regulatory network of the cells and on the other hand calls for detailed investigation of the p53 modulators. Unpublished data from our laboratory suggest that the Δ133p53 isoform is highly prone to aggregation. It can aggregate in vitro, without any adjuvant, significantly faster than FLp53. Whether this isoform can co-aggregate with FLp53 remains to be seen. It is plausible that in vivo aggregation of the Δ133p53 isoform is due to its over-expression. Based on the current knowledge it can be speculated that the N-terminal p53 isoforms can inactivate p53 by co-aggregation.

## 5. Evolutionary Origin

**Figure 3:**
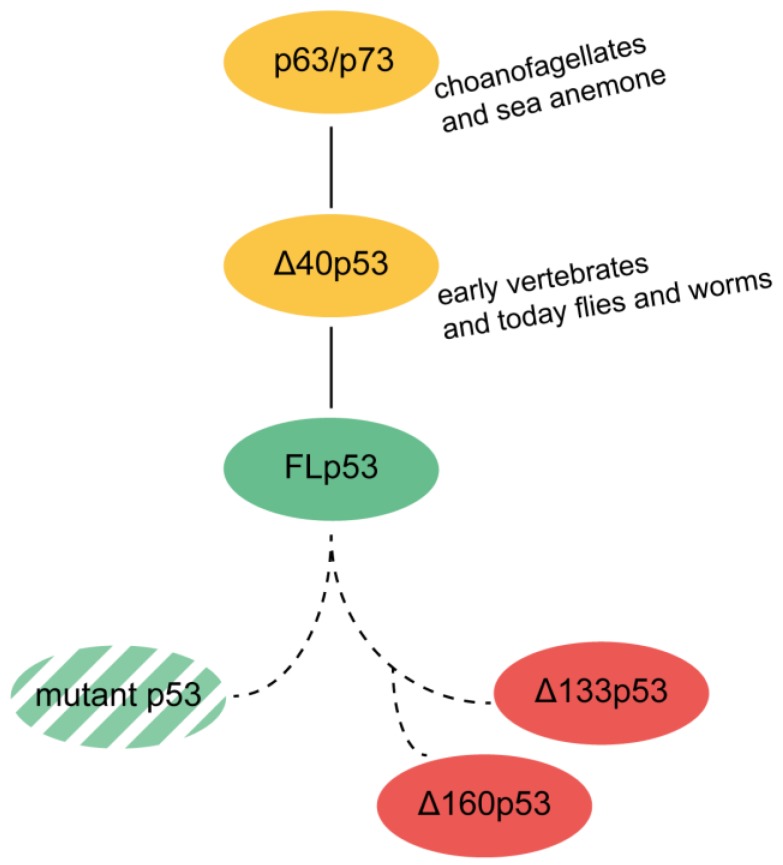
Possible evolutionary pathway of the p53 protein as a germline surveillance factor and its isoforms. p63/p73 genes evolved first in the early multicellular organisms from p53, similar to how the ∆40p53 isoform evolved in primitive vertebrates. From this, FLp53 evolved in higher organisms. From FLp53, various mutant p53 isoforms as well as the N-terminal truncated isoforms ∆133p53 and ∆160p53 developed.

The origin of p53 lies in a gene more similar to a combined p63/p73 gene (Figure 3) found in today’s single-cell choanoflagellates [126] and sea anemone [127]. Their initial task was to maintain the germline under starvation and under the conditions of DNA damage [128].

This precursor gene duplicates to create a p53 gene with the advent of somatic stem cells or progenitor cells, which are able to renew tissues and organs [129]. p53 addresses the requirements of an organism to live a certain time until it reaches reproductive maturity [130]. Vertebrates require a longer time to attain maturity and hence, require a plan for self-renewal. Human p53 is located in somatic tissue and the germline, where it maintains the balance between apoptosis and regeneration. In this context, p53 works as a tumor repressor. For adult worms, or flies, p53 is not needed for somatic cells. There, it only expresses in the germline cells at the conditions of starvation or DNA-damage [128]. The early p53 gene probably resembled one of today’s isoforms, ∆40p53 [50], as p53 homologs in lower organism are closer to ∆40p53. This isoform cannot transactivate genes which control cell proliferation and growth [45,50]. ∆40p53 interacts with FLp53; together they suppress cancerous growth [131,132], but ∆40p53 alone is tumorigenic [133]. Because ∆40p53 would have no effect on the cells of post mitotic organisms, it seems plausible that ∆40p53 is the predecessor of the FLp53 [50]. Hence, FLp53 might have developed to fine-tune the early ∆40p53 pathway.

The issue is then as to when the even shorter isoforms ∆133p53 and ∆160p53 evolved. Since the FLp53 evolved with the onset of tissue homeostasis, we could speculate that the isoforms ∆133p53 and ∆160p53 evolved much later for maintenance of the somatic cells with DNA damage, practically for the benefit of the whole organism. Further investigations are required to draw a conclusion on the origin of the ∆133p53 and ∆160p53 isoforms (Figure 3).

## 6. Conclusions/Summary/Key Points

The p53-network governs the cell cycle and proliferation [4] in the demand of a multicellular organism with multigeneration tissues. p53 has evolved from a precursor protein to maintain the genomic stability of firstly the germline [128], and then the whole organism. Dysfunction of the p53 network can cause cancer. Understanding the principles of the p53 and its isoforms will enrich our knowledge and help in the prognosis and treatment of cancer. p53 research has focused on mutation in the *TP53* gene as the major cause for cancer [38]. Studies have shown that isoform expression is independent of *TP53* mutation status [64,117,134] and that the mutation status alone is not enough to predict cancer progression [117].

**Figure 4:**
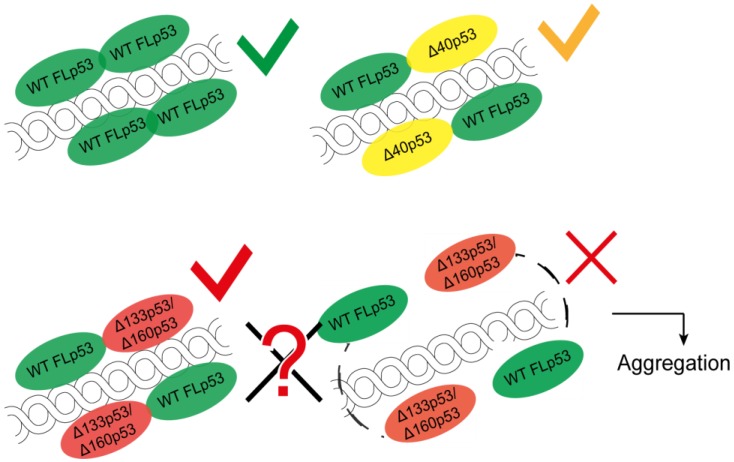
Possible modes of action of the p53 isoforms with respect to gene activation. Binding of FLp53-homotetramers to DNA response elements leads to activation of the standard gene repertoire. Binding of ∆40p53-FLp53 heterotetramers leads to activation of an alternative set of genes. However, these heterotetramers are not inactive. From the current data it is not known whether the isoforms ∆133p53 or ∆160p53 can bind to DNA and induce transcription. Even if they do so the possibility that the same DNA/genes will be targeted is low. Even so, the binding could occur in a much more complex manner as depicted in the figure. We hypothesize that these isoforms inactivate FLp53 when tetramerizes with it. They can also lead to aggregation and degradation. The ∆133p53 and ∆160p53 p53 isoforms may inactivate the p53 pathway by a ‘dominant negative effect’ or by ‘gain of function’.

As discussed before, the gene encoding p53, *TP53*, can translate 12 different isoforms by alternative promoter usage, alternative splicing and by the presence of multiple IRES [13,14]. These isoforms have varying N- and C-termini and show different effects on p53 function. The ∆40p53 isoform is similar to the FLp53 and causes no inhibitory action leading to apoptosis (Figure 4) [47,49]. On the contrary, ∆133p53 and ∆160p53 act more like a survival factor [59,60,135] and could lead to a more resistant cancer [63]. It is unclear whether these two isoforms with truncated DBD are capable of binding to the specific DNA sequences. However, owing to the arginine/lysine-rich CTD, these isoforms may bind to DNA non-specifically. It is also not known whether these isoforms can activate gene expression by forming usual tetramers. Due to the intact OD, they can potentially form tetramers with themselves and also with FLp53, thereby inhibiting the regular p53 pathway (Figure 4). ∆160p53 has even been found as responsible for a gain of function which is usually exhibited by mutant p53s [63].

The strong propensity of p53, its mutants, and isoforms to aggregate [57,99,136] hints that there might be other mechanisms for inactivating p53 (Figure 4). The truncations (Figure 2C,D) of the isoforms ∆133p53 and ∆160p53 could be responsible for their aggregation property. Conversely, the truncated isoforms assembled with FLp53 or with themselves could activate a different set of REs by dominant negative effect or gain of function. These would also disrupt the usual p53 pathway (see Table 1).

Many ambiguities still remain: How do the shorter isoforms ∆133p53 and ∆160p53 interact with the FLp53? How do point mutations interfere with the p53 network and its isoforms? How much does the effect of mutated p53 contribute to the effect of the short isoforms? Understanding the p53 isoforms with molecular mechanisms will be the next big objective in cancer research.

## Figures and Tables

**Table 1:** p53 isoforms: their cellular effects and appearance in various cancer types.

p53 Isoforms with Description		Discovered and Reported Cellular Effects	Cancers with Appearance or Overexpression
FLp53β	Lacks 52 amino acids from the C-terminus of FLp53; devoid of OD and CTD	Promotes senescence [113] loss of expression in breast cancer [39]	Colorectal cancer [52] Renal cell carcinoma [114] Acute myeloid leukemia (AML) [40] Loss of expression in breast cancer [39] Squamous cell carcinoma [115] Expressed only melanoma, not in melanocytes [116]
FLp53γ	47 amino acid truncated from the C-terminus; devoid of an OD or CTD	Cytotoxic [58] Patients with p53γ have an overall good survival rate, along with a low cancer reoccurrence, and without p53γ a particularly poor prognosis [58]	Breast cancer (good prognosis) [64] Acute myeloid leukemia (AML) (overexpression after chemotherapy followed by higher overall survival) [40] Shorter progression-free survival in uterine serous carcinoma [117]
Δ40p53	N-terminal truncated p53, starts from Met 40; lacks TAD I	Induces cell death [46] Prolongs pluripotency [46] Decreases pancreatic-β-cell proliferation and regulates glucose homeostasis [46] tumor suppression [118], and balance with tissue regeneration [50] regulates folding, oligomerization, and post-translational modification of p53 complexes [4]	Mucinous ovarian cancer [119] glioblastoma multiforme [120] Serous ovarian cancer [121] Expressed in melanoma, but not in melanocytes; and improved survival in ovarian cancer [116] Li-Fraumeni syndrome [122]
Δ133p53α	Lacks 132 amino acids from the N-terminus; lacks TAD I, TAD II, the proline-rich region (PRD), and part of the DBD	Pro-survival factor [9] Inhibits senescence [113], p53 mediated apoptosis [39], and p53 transcriptional activity [58] Extends cellular replicative life spans in human fibroblasts [113] Delays the onset of replicative senescence [60] May rescue embryogenic cells from radiation induced apoptosis [55]	Colorectal cancer [52] Serous ovarian cancer [121] Overexpression in primary breast cancer [39] Cholangiocarcinoma with shortened overall survival [123]
Δ133p53β	Lacks 132 amino acids from NTD including TAD I, TAD II, PRD, parts of DBD. Is also devoid of OD, and CTD as other β forms	Promotes epithelial–mesenchymal transition in breast cancer cells [124] Fosters cancer stem cell potential [125]	Enhances cancer cell stemness in breast cancer [125] Expressed in melanoma cell lines [116] Increased breast cancer pervasiveness [124]
Δ160p53	Truncated at N-terminus, starts with Met 160; lacks TAD I, TAD II, PRD, and parts of the DBD	Bears pro-oncogenic traits [63] can induce mutant-like phenotypes [63] probably widely involved in oncogenic mutant p53 gain of functions [63]	Unknown to date
